# Spontaneous Transformation of Murine Oviductal Epithelial Cells: A Model System to Investigate the Onset of Fallopian-Derived Tumors

**DOI:** 10.3389/fonc.2015.00154

**Published:** 2015-07-17

**Authors:** Michael P. Endsley, Georgette Moyle-Heyrman, Subbulakshmi Karthikeyan, Daniel D. Lantvit, David A. Davis, Jian-Jun Wei, Joanna E. Burdette

**Affiliations:** ^1^Department of Medicinal Chemistry and Pharmacognosy, College of Pharmacy, University of Illinois at Chicago, Chicago, IL, USA; ^2^Department of Pathology, Northwestern University, Chicago, IL, USA

**Keywords:** carcinogenesis, fallopian tube, cancer, ovarian cancer, and model system

## Abstract

High-grade serous carcinoma (HGSC) is the most lethal ovarian cancer histotype. The fallopian tube secretory epithelial cells (FTSECs) are a proposed progenitor cell type. Genetically altered FTSECs form tumors in mice; however, a spontaneous HGSC model has not been described. Apart from a subpopulation of genetically predisposed women, most women develop ovarian cancer spontaneously, which is associated with aging and lifetime ovulations. A murine oviductal cell line (MOE^LOW^) was developed and continuously passaged in culture to mimic cellular aging (MOE^HIGH^). The MOE^HIGH^ cellular model exhibited a loss of acetylated tubulin consistent with an outgrowth of secretory epithelial cells in culture. MOE^HIGH^ cells proliferated significantly faster than MOE^LOW^, and the MOE^HIGH^ cells produced more 2D foci and 3D soft agar colonies as compared to MOE^LOW^ MOE^HIGH^ were xenografted into athymic female nude mice both in the subcutaneous and the intraperitoneal compartments. Only the subcutaneous grafts formed tumors that were negative for cytokeratin, but positive for oviductal markers, such as oviductal glycoprotein 1 and Pax8. These tumors were considered to be poorly differentiated carcinoma. The differential molecular profiles between MOE^HIGH^ and MOE^LOW^ were determined using *RNA-Seq* and confirmed by protein expression to uncover pathways important in transformation, like the p53 pathway, the FOXM1 pathway, WNT signaling, and splicing. MOE^HIGH^ had enhanced protein expression of c-myc, Cyclin E, p53, and FOXM1 with reduced expression of p21. MOE^HIGH^ were also less sensitive to cisplatin and DMBA, which induce lesions typically repaired by base-excision repair. A model of spontaneous tumorogenesis was generated starting with normal oviductal cells. Their transition to cancer involved alterations in pathways associated with high-grade serous cancer in humans.

## Introduction

Epithelial ovarian cancer (EOC) has a disproportionately high-mortality rate in comparison to other gynecologic malignancies, ranking first overall (1). The American Cancer Society estimates that 14,180 women will succumb to this disease annually and 21,290 will be newly diagnosed in 2015 (2). EOC can be divided into five common histotypes including mucinous, endometrioid, clear cell, low-grade serous, and high-grade serous carcinoma (HGSC) (3). HGSC is the most prominent EOC histotype and accounts for nearly 75% of all EOC-related mortality. Moreover, the 5-year survival rate from 2003 to 2009 was 44%, a mere 8% improvement from the mortality rates observed 30 years earlier (1). The underlying molecular events contributing to initiation and progression are still lacking, leading to patients rarely being diagnosed in stage I (4). Aspects contributing to this high-mortality rate are the debatable cellular origin of the disease and the paucity of models representing spontaneous tumor formation (5).

The source of EOC is currently being investigated with both the ovarian surface epithelia (OSE) and the fallopian tube secretory epithelial cell (FTSEC) serving as possible progenitor populations (6). The ovarian surface was considered the source of EOC for decades with recent evidence pointing to the fallopian tube or both. For example, women undergoing prophylactic salpingo-oophorectomies due to germ-line mutations in either *BRCA1* or *BRCA2* provided pathologists with tissue specimens that contained neoplastic lesions in the fimbriae of the fallopian tubes (7). Fallopian tube lesions occurred in women with HGSC and with serous tubal epithelial carcinomas (STICs), leading to the hypothesis that the fallopian tube secretory epithelium is a potential source of HGSC (8). Recent evidence specifically demonstrates that the secretory epithelial cells of the fallopian tube (or oviduct in mice) are the source of tubal-derived HGSC (9–11).

There are many well-established cellular and animal models used to study FTSEC or oviductal epithelial cell transformation by using SV40, which functionally inhibits both the Rb and p53 tumor-suppressor pathways (12–15). The concern with using SV40 is that blocking these two important tumor-suppressor pathways as well as overexpressing oncoproteins, such as c-Myc or H-Ras, will induce tumorigenesis in many, if not all cell types and that mutation and loss of p53 function are not equivalent (16). Mutation in p53 is the defining event that is common to 96–100% of HGSC (17). Genetically modified models have been generated from both the OSE and FTSECs (18, 19) generally targeting the specific genes associated with human HGSC (18, 19). This targeted approach to tumorigenesis has provided important evidence that both OSE and oviductal cells (murine equivalent of fallopian tube) can form tumors, but lacks information regarding the mechanisms for these changes to occur. Furthermore, in human disease, it is unclear whether these commonly mutated genes are normally involved in disease initiation and/or progression.

The development of spontaneous models of HGSC would assist in understanding the origins and progression of this disease. For decades, the OSE was the primary cell type thought to give rise to HGSC with numerous cellular and transgenic animal models supporting this theory (20–24). For example, both rat and murine OSE cells isolated and passaged in culture have provided evidence that the OSE can give rise to tumors that exhibit many phenotypic and genetic similarities to the human disease (25–28). Another critical model of spontaneous ovarian cancer is the laying hen, which mainly develops endometrioid EOC, instead of HGSC, and has a unique oviduct that primarily functions as a shell gland (29–31). Therefore, this study focused on the development of a new spontaneous model of ovarian cancer derived from outbred CD1 oviducts. Similar to OSE models, continuous passaging produced transformed cells that demonstrated unique changes in transcription consistent with the human disease. This model provides a unique tool for understanding aspects of tumorigenesis from oviductal cells. This model may also be used to understand how cells develop resistance to chemotherapy.

## Materials and Methods

### Animals

Female CD1 mice were purchased from Harlan (Indianapolis, IN, USA) and NCr *nu/nu* athymic (nude) female mice from Taconic (Hudson, NY, USA). Animals were treated in direct compliance with the National Institutes of Health (NIH) Guide for the Care and Use of Laboratory Animals, using protocols approved by the Animal Care Committee at the University of Illinois at Chicago (UIC). Mice were housed in a temperature and light controlled environment (12 h light, 12 h dark) and provided food and water *ad libitum*. Nude mice were euthanized when tumors developed and based on loss of wellness and humane endpoints. All mice were euthanized by CO_2_ asphyxiation followed by cervical dislocation.

### Isolation of murine oviductal epithelial cells

Primary murine oviductal epithelial (MOE) cells (also called murine tubal epithelial cells or MTEC) were pooled from multiple oviducts to establish one cell line from 8-week-old female CD1 mice, as previously described (32). To ensure purity, oviducts were carefully excised, and using a dissecting scope, removed the ovaries, bursa, uterus, and fat pads. MOE cells were maintained in complete medium containing α-MEM modified Eagle’s medium (CellGro, Manassas, VA, USA) supplemented with ribonucleosides, deoxynucleosides, and l-glutamine and supplemented with 10% fetal bovine serum, 2 mM l-glutamine (Life Technologies, Grand Island, NY, USA), 2 μg/ml epithelial growth factor, 5 μg/ml insulin, 5 μg/ml transferrin, 5 ng/ml sodium selenite (Roche; Indianapolis, IN, USA), 1 mg/ml gentamycin (CellGro; Manassas, VA, USA), and 18.2 μg/ml β-estradiol (Sigma Aldrich; St Louis, MO, USA). MOE cells were maintained in a humidified atmosphere containing 5% CO_2_ at 37°C.

Once the primary isolated MOE cells became established in culture, the MOE^LOW^ cells (passages 8–25) were continuously passaged to generate the experimentally aged, MOE^HIGH^ cells (passages 85–120). The MOE cells were verified to be clean of *Mycoplasma* spp. and 12 other rodent pathogens after analysis using the IMPACT III Profile test (IDEXX BioResearch, Columbia, MO, USA).

### Western immunoblotting

Murine oviductal epithelial cells were lysed in modified RIPA buffer and protein concentration was determined, as previously described (33). Whole cell lysates in RIPA buffer (30 μg/well) were separated, transferred to nitrocellulose membrane (GE Healthcare Life Sciences, Pittsburgh, PA, USA), blocked, and incubated with primary antibody shaking at 4°C overnight. Membranes were incubated with HRP-conjugated secondary antibody and exposed to SuperSignal West Femto substrate (Thermo Scientific, Waltham, MA, USA). All blots were photographed using the FluorChem E documentation system (Protein Simple; Santa Clara, CA, USA). Densitometry is calculated from three blots.

Primary antibodies include the following: phosphorylated-Rb1 (Ser780), c-myc, p53, E-cadherin, and α-tubulin were all obtained from Cell Signaling Technology, Inc. (Danvers, MA, USA); cyclin E1, N-cadherin, and OVGP1 were from Abcam (Cambridge, MA, USA); p21, p16^INK4A^, and FoxM1 from Santa Cruz Biotechnologies (Santa Cruz, CA, USA); and PAX8 from Proteintech (Chicago, IL, USA).

### MOE cell RNA preparation and sequencing using next-generation RNA-sequencing technology

MOE^LOW^ (passages 8, 9, and 10) and MOE^HIGH^ (passages 90, 103, and 113) cells were plated and at 75% confluency, they were washed with PBS, lysed, and total RNA extracted in Trizol reagent (Life Technologies), according to manufacturer’s protocol. To ensure that there was no residual genomic DNA contamination, samples were treated with RNase-free DNase I and RNA was further purified using RNeasy Mini cleanup (Qiagen Inc., Valencia, CA, USA). The concentration of the total RNA was determined using the NanoVue Plus spectrophotometer provided by the UIC Resource Research Center Core (GE Healthcare Bio-Sciences, Piscataway, NJ, USA) and RNA purity as determined by the RNA integrity number (RIN) was verified using TapeStation 2200 (Agilent Technologies, Palo Alto, CA, USA).

Each total RNA sample had ribosomal RNA removed using TruSeq Stranded Total RNA with Ribo-Zero (Illumina, San Diego, CA, USA). Strand-specific libraries were constructed and quantitated using Qubit, and cDNAs verified by qPCR. The resulting six libraries were pooled and sequenced using TruSeq SBS sequencing kit 3 for 101 cycles on a HiSeq2000 (Illumina) and processed with Casava (version 1.8.2.), according to the manufacturer’s protocol.

### RNA-seq transcriptome analysis

The quality of DNA reads (fastq format) was evaluated using FastQC. The data were analyzed following the previously described procedure (34). Briefly, the reads were aligned to the *Mus musculus* genome (mm10) using TopHat, version 2.0.8b^1^. Aligned reads were used to determine the expression of known mmu10 gene annotations from the University of California-Santa Cruz website using Cufflinks version 2.1.1^2^. The individual transcript files generated by Cufflinks were merged into a single gene annotation file and used to determine the differential expression analysis feature in Cuffdiff.

Differential expression was determined to be statistically significant by Cuffdiff using a false discovery rate (FDR)-adjusted *p*-value, where significance was set to *p* ≤ 0.05 (35). Differential expression analysis was processed using R-package (cummeRbund) and used to compare the MOE^LOW^ and MOE^HIGH^ cells. The cummeRbund results were used to create a dendrogram comparing the differences between all MOE^LOW^ and MOE^HIGH^ cell passages. Furthermore, pathway analysis was performed on transcript lists from both cell lines using GeneCoDis to identify the KEGG and Panther pathways that are significantly different between MOE^LOW^ and MOE^HIGH^ cell lines (36–38). RNA-sequencing technology (RNA-seq) and data analysis were supported by the Northwestern University Next-Generation Sequencing Core Facility and Dr. Matthew Schipma.

### Quantitative PCR analysis

Total RNA (1.0 μg) was reverse transcribed using the iScript cDNA synthesis kit (BioRad), according to manufacturer’s instructions. cDNA was amplified and analyzed using ViiA7 iCycler real-time SYBR PCR detection system (Life Technologies). Samples are normalized relative to the internal control *Gapdh* and MOE^HIGH^ cells were compared to the MOE^LOW^ cells (ΔΔC_t_ expressions) of *Cdkn1a*, *Foxm1*, *Trp53*, *Ovgp1*, and *Pax8*.

### Cell proliferation

Sulforhodamine B (SRB) assays were used to determine proliferation of MOE cells, as described previously (33). Briefly, MOE cells (1.0 × 10^3^/well) were incubated for 0, 12, 24, 48, and 72 h. Absorbance was measured at 505 nm using a BioTek Synergy 2 microplate reader (BioTek). All assays were repeated in at least three biological replicates. For experiments using doxorubicin, cisplatin, paclitaxel, and DMBA, cells were treated with eight concentrations in four independent experiments and the data plotted represent the average cell viability for each concentration. The values of LC_50_ were determined from the averaged data that were fit to the curve using Prism.

### Clonogenic assay

MOE^LOW^ and MOE^HIGH^ cells were plated (200 cells/60 mm-dish) and incubated for 6 days. After incubation, cells were washed, fixed with 4% (*w/v*) paraformaldehyde, and stained with 0.04% (*w/v*) crystal violet for 30 min at room temperature. Plates were washed with deionized H_2_O, dry, and photographed using the FluorChem E documentation system (Protein Simple, Santa Clara, CA, USA). Numbers of foci were analyzed using Image J software (NIH) from four biological replicates.

### Soft agar colony formation assay

Murine oviductal epithelial (1.5 × 10^4^) cells were suspended in 0.35% (*w:v*) agar on top of a 0.5% (*w:v*) base agar layer (39). Cells were incubated for 14 days with media changed every 3 days. Final colonies were imaged on a Nikon Eclipse TS100 microscope and colonies were counted using Image J software from three biological replicates.

### Tumorgenicity in nude mice

The procedure of injections was performed, as previously described (13). Briefly, both *s.c*. (1.0 × 10^6^ cells in Matrigel/animal) and *i.p*. (1.0 × 10^7^ cells in PBS/animal) injections were done into all mice (*n* = 5 mice/group). Once palpable, tumor growth was monitored weekly using digital calipers. Animals were sacrificed at humane endpoints and tumors were excised, weighed, and either embedded in paraffin for immunohistochemistry (IHC) or flash frozen in liquid nitrogen for protein and mRNA analysis. Additionally, gross internal anatomy was inspected for any signs of *i.p*. disease.

### Immunohistochemistry

Paraffin-embedded tissues sectioning, H&E staining, and IHC were completed, as described previously (40). Tissue sections were incubated with the following primary antibodies overnight at 4°C: anti-CK8 (1:200; Developmental Studies Hybridoma Bank, Iowa City, IA, USA); anti-Pax8 (1:200; Proteintech, Chicago, IL, USA); anti-Ki67 and anti-OVGP1 (1:100; Abcam, Cambridge, MA, USA). DAB staining was used to detect protein expression in tissue sections. Stained sections were analyzed by a pathologist (Dr. Jian-Jun Wei, Northwestern University). Slides were imaged on a Nikon Eclipse E600 microscope using the 40× objective.

### Immunofluorescence

Murine oviductal epithelial (5.0 × 10^4^ cells/well) cells were plated in an eight-well chamber slide and stained for various proteins, as previously described (41). Briefly, cells were fixed in 4% (*w/v*) PFA, permeabilized with 0.2% (*v/v*) Triton X-100, blocked with 10% (*v/v*) goat serum, incubated with primary antibody for 1 h at room temperature. The primary antibodies used were anti-Pax8 (1:200; Proteintech, Chicago, IL, USA); and anti-OVGP1 (1:200; Abcam, Cambridge, MA, USA). Proteins were visualized by incubation with AlexaFluor488 (green) secondary antibodies and slides were mounted using Vectashield Mounting Medium containing DAPI (Vector Laboratories; Burlingame, CA, USA). Slides were imaged on a Nikon Eclipse E600 microscope and captured using Nikon DigitalSight DS-Ri1 camera.

### Statistical methods

Statistical analysis was carried out using GraphPad Prism software (GraphPad Software, La Jolla, CA, USA). All values are expressed as the means of samples ± SEM. Statistical significance was determined by unpaired *t*-test a *p* < 0.05 was considered to be statistically significant.

## Results

### MOE^HIGH^ demonstrate *In vitro* and *In vivo* evidence of spontaneous cellular transformation

**Figure 1:**
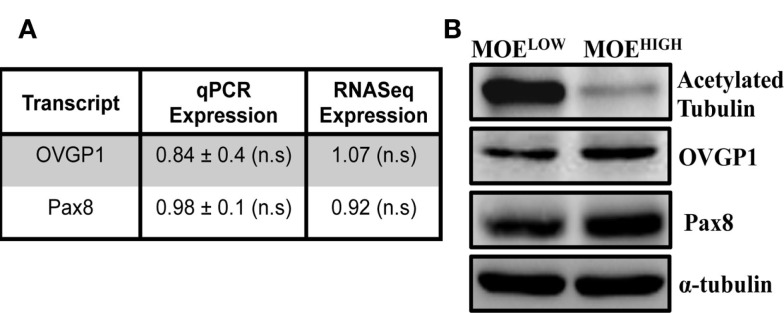
**MOE^HIGH^ lose acetylated tubulin upon serial passaging**. **(A)** OVGP-1 and Pax8 expression levels were similar in qPCR and RNA sequencing and not significant (n.s.) between MOE^HIGH^ and MOE^LOW^. **(B)** Western blotting on MOE^HIGH^ and MOE^LOW^ cell lysates probed for acetylated tubulin, OVGP-1, PAX8, and normalized to α-tubulin.

In order to derive an oviductal cell line, CD1 mice were utilized as a source of primary cells because they are an outbred strain that might provide a model for human disease. Murine cells were used to compare to existing ovarian surface models, and because they typically can be immortalized on plastic without the use of viral oncoproteins. CD1 cells were confirmed to be of epithelial origin, as previously described (32). The CD1 cells were then continuously cultured to up to 130 passages to generate two cell models, MOE^LOW^ (passages 5–25) and MOE^HIGH^ (passages 85–120). Both MOE^LOW^ and MOE^HIGH^ expressed oviductal glycoprotein 1 (OVGP1) and PAX8 based on RNA (Figure 1A) and protein expression (Figure 1B; Figure S1 in Supplementary Material) (42, 43). MOE^HIGH^ cells lost acetylated tubulin and expressed Pax8, consistent with other oviductal models cultured on plastic that tend to undergo senescence and lose cilia function that produce an outgrowth of the secretory subtype of epithelium. Recent evidence suggests that most HGSC originate from the FTSECs (44). The outgrowth of secretory cells in fallopian tube fimbria is often described by pathologists as a putative preneoplastic lesion called a SCOUT (11).

**Figure 2:**
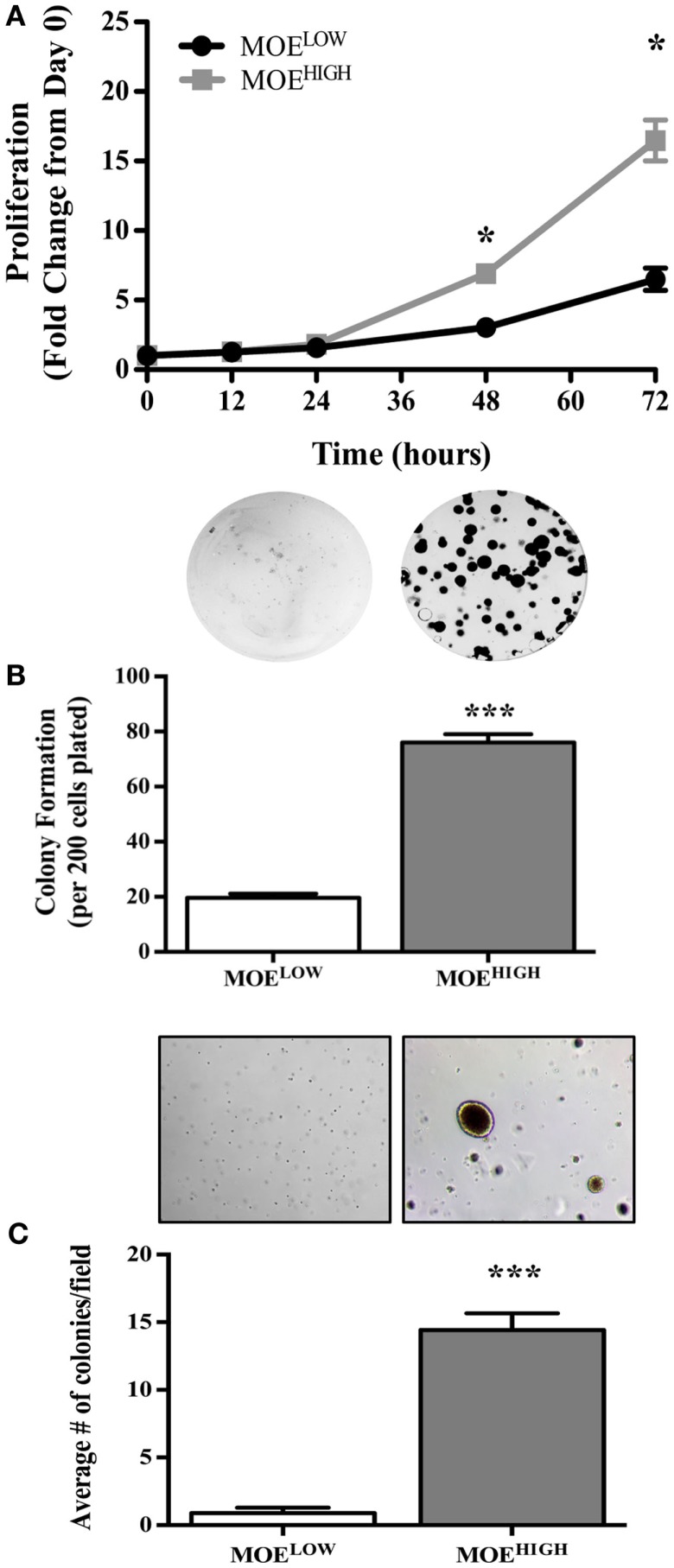
**MOE^HIGH^ cells demonstrate *in vitro* aspects of transformation, such as enhanced proliferation, foci formation, and soft agar colony growth, as compared to MOE^LOW^**. **(A)** Proliferation of MOE^HIGH^ and MOE^LOW^ cells was analyzed using SRB assay and data calculated as the fold change from day 0. **(B)** MOE^HIGH^ and MOE^LOW^ cells were tested for 2D colony formation after 6 days using a clonogenic assay. **(C)** MOE^HIGH^ and MOE^LOW^ cells were analyzed for soft agar colony formation after 14 days. *p-value >0.01.

MOE^HIGH^ cells were tested for *in vitro* aspects of transformation. First, the rate of proliferation of MOE^HIGH^ was compared to MOE^LOW^. MOE^HIGH^ cells proliferated significantly more than MOE^LOW^ after 48 and 72 h (Figure 2A). The average doubling time of MOE^HIGH^ cells was 17.3 h as compared to the MOE^LOW^ doubling time of 24.1 h. Next, a clonogenic assay was employed to monitor the ability of MOE^HIGH^ cells to form colonies as compared to MOE^LOW^. MOE^HIGH^ cells formed significantly more clonogenic colonies than MOE^LOW^ after 6 days (Figure 2B). MOE^HIGH^ demonstrated on average 76 ± 3 colonies or about 38% of the cells plated formed foci as compared to MOE^LOW^, where only 9.8% formed foci. Finally, the cells were grown in soft agar to test whether they exhibited anchorage independent growth. MOE^HIGH^ cells formed a significant number of soft agar colonies (14 ± 1) after 14 days, while MOE^LOW^ did not form colonies and persisted as single cells in agar (Figure 2C).

**Figure 3:**
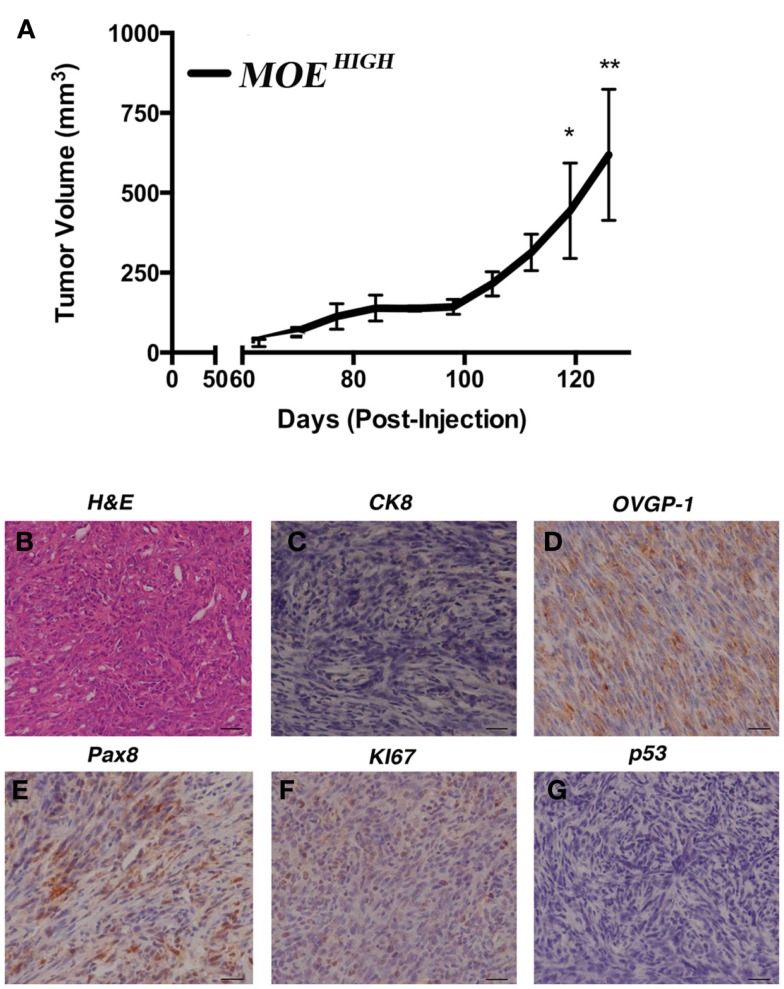
**MOE^HIGH^ cells undergo spontaneous transformation and form tumors**. **(A)** Athymic female nude mice were injected subcutaneously and intraperitoneally with MOE^HIGH^ and MOE^LOW^ cells. MOE^HIGH^ formed subcutaneous tumors but not intraperitoneal explants. MOE^LOW^ cells failed to form any tumors. Tumor volume was measured with calipers. Hematoxylin and Eosin staining **(B)** and immunohistochemistry **(C–G)** performed on s.c. MOE^HIGH^ tumors. Tumors were malignant, undifferentiated, and mesenchymal and were negative for CK8 **(C)**. Tumors were of oviductal origin staining positive for OVGP1 **(D)** and Pax8 **(E)**. Tumors were highly proliferative based on positive Ki67 **(F)** and did not demonstrate stabilized p53 **(G)**. Scale bars = 100 μm.

In order to confirm whether the *in vitro* characteristics of transformation identified in MOE^HIGH^ contributed to tumor formation, female athymic nude mice (*n* = 5 MOE^HIGH^ and MOE^LOW^) were injected subcutaneously with 1 × 10^6^ cells and intraperitoneally with 1 × 10^7^ cells. All of the mice injected with MOE^HIGH^ cells developed subcutaneous tumors that grew over time and ultimately mice were sacrificed for humane endpoints after an average of 117 ± 9 days (Figure 3A). MOE^HIGH^ cells failed to develop any tumors when injected into the peritoneal space. MOE^LOW^ failed to develop tumors in either the s.c. or i.p. sites. Subcutaneous tumors derived from MOE^HIGH^ cells were evaluated by a pathologist based on morphological features from H&E staining as well as using a variety of immunohistochemical stains. Tumors were solid with spindle-like structures and were highly cellular with moderate nuclear atypia and abundant mitosis (Figure 3B). The morphology was sarcoma like with infiltration of the tumor identified in the skeletal muscle. The tumors were highly mesenchymal exhibiting little or no cytokeratin 8 (Figure 3C) positivity or WT1 staining (not shown). OVGP1 and Pax8 are markers of Müllerian origin, and PAX8 is used to classify tumors as the serous histotype. Tumors were OVGP1 positive (Figure 3D), demonstrated some Pax8 positivity, but it was both nuclear and cytoplasmic as well as sporadic (Figure 3E). A high percentage of tumor cells stained with Ki67 indicating increased proliferation (Figure 3F). Tumors did not express stabilized p53 (Figure 3G). Overall, the tumors were considered poorly differentiated spindle cell neoplasia of oviductal origin.

### Transcriptome of MOE^HIGH^ reveals changes responsible for transformation

**Figure 4:**
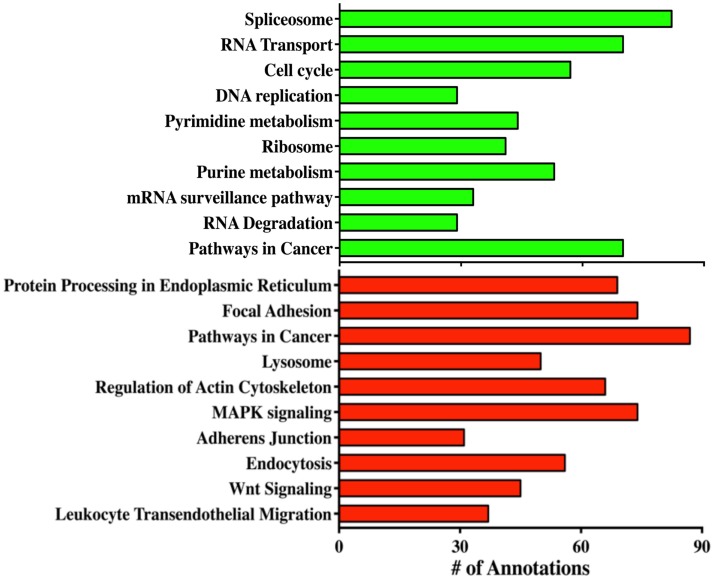
**RNA sequencing reveals biological processes altered in MOE^HIGH^ cells**. Gene ontology analysis of the pathways that were altered in MOE^HIGH^ cells compared to MOE^LOW^. Green bars represent pathways that were upregulated and red bars indicate the downregulated pathways.

In order to reveal some of the pathways that might contribute to tumor formation of MOE^HIGH^ cells, RNA sequencing was performed on three different passages of MOE^LOW^ (8, 9, 10) as compared to MOE^HIGH^ (90, 103, 113). Samples were barcoded and sequenced using Illumina HiSeq2500 sequencing. Samples clustered based on passage number using a dendrogram analysis (Figure S2 in Supplementary Material). The top 20 most significantly up- and down-regulated transcripts are shown in Table 1. Pathway analysis revealed that the most highly upregulated biological processes involved the splicesome, RNA transport, the cell cycle, and DNA replication (Figure 4). Biological processes that were repressed included processing in the endoplasmic reticulum, focal adhesion, pathways in cancer, and the lysosome.

**Table 1:** **Table showing the list of genes that were significantly regulated in MOE^HIGH^ cells from RNA sequencing analysis**.

	Gene name	RefSeq ID	RPKM 1	RPKM 2	Log_2_ fold change	FDR-adjusted *p*-value	Fold change
1	Adam19	NM_009616	16.81	0.01	−10.73	0.033	1698.92
2	Fbln5	NM_011812	16.12	0.01	−10.65	0.0397	1612.29
3	Gpc4	NM_008150	73.1	0.05	−10.47	0.0002	1421.01
4	Aldh1a2	NM_009022	99.25	0.07	−10.47	0.001	1413.93
5	Meg3, Mir1906-1, Mir770	NR_030427	35.16	0.03	−10.2	0.0005	1176.35
6	Myl9	NM_172118	692.44	0.59	−10.2	0.0002	1175.7
7	Igfbp2	NM_008342	411.81	0.39	−10.06	0.0002	1066.45
8	Bgn	NM_007542	194.46	0.2	−9.91	0.0002	961.53
9	Dkk3	NM_015814	145.8	0.16	−9.83	0.0002	909.81
10	Rcn3	NM_026555	201.24	0.24	−9.73	0.0002	851.81
11	Ar	NM_013476	6.72	0.01	−9.7	0.0172	833.48
12	Acta2	NM_007392	4445.02	5.73	−9.6	0.0002	775.58
13	Gpx3	NM_008161	101.12	0.14	−9.54	0.0002	745.5
14	Lox	NM_010728	92.18	0.13	−9.46	0.0002	706.53
15	Col1a2	NM_007743	133.43	0.19	−9.46	0.0002	702.45
16	Luzp2	NM_178705	11.05	0.02	−9.43	0.0264	689.72
17	Tagln	NM_011526	4014.57	6.11	−9.36	0.0002	656.56
18	Thbd	NM_009378	12.43	0.02	−9.36	0.0452	656.18
19	Itih2	NM_010582	43.31	0.07	−9.36	0.0002	655.09
20	Rian	NR_028261	34.51	0.06	−9.29	0.0002	626.01
21	Wnt7b	NM_009528	0.08	36.78	8.77	0.00017	436.14
22	Gsta1	NM_008181	0.08	31.37	8.66	0.00648	403.5
23	Myo16	NM_001081397	0.01	2.83	8.42	0.01787	342.22
24	Ccnb1ip1	CUFF.5283.1	0.2	44.25	7.81	0.00017	224.45
25	Slco2a1	NM_033314	0.13	29.46	7.77	0.00017	218.98
26	Adh7	NM_009626	0.47	102.2	7.77	0.00017	218.97
27	Cdh16	NM_001252627	0.35	50.13	7.17	0.00017	144.34
28	Mrc1	NM_008625	0.1	12.8	6.94	0.00017	122.91
29	Aqp5	NM_009701	0.51	60.08	6.88	0.00017	117.65
30	Nup210	NM_018815	0.05	4.97	6.78	0.00017	109.99
31	Upk1b	NM_178924	0.65	69	6.73	0.00017	106.28
32	Arhgef26	NM_001081295	0.02	2.1	6.64	0.00105	99.51
33	Itgb2	NM_008404	0.43	42.78	6.63	0.00017	99.26
34	Crb2	NM_001163566	0.16	15.21	6.56	0.00017	94.38
35	Ppl	NM_008909	0.53	45.97	6.45	0.00017	87.52
36	Prl2c2	NM_031191	1.96	168.62	6.43	0.00017	86.23
37	Ano1	NM_001242349	0.47	39.5	6.39	0.00017	84
38	Fmnl1	NM_019679	0.14	11.08	6.3	0.00017	78.76
39	Cxcl5	NM_009141	0.09	6.83	6.29	0.00223	78.09
40	Mgst2	NM_174995	0.38	28.35	6.22	0.00062	74.59

*Genes from 1 to 20 were downregulated and genes from 21 to 40 were upregulated*.

### MOE^HIGH^ cells harbor splice variant of p53 with differential action on target genes

The cancer genome atlas helped to define critical pathways altered in high-grade serous tumors (17). In order to compare MOE^HIGH^ to human tumor samples, pathways implicated in human disease were interrogated. RNA sequencing revealed that p53 in MOE^LOW^ and MOE^HIGH^ cells was not mutated; however, MOE^HIGH^ cells had a significant upregulation of a splice variant of p53 that has the last 26 amino acids eliminated and has 16 unique residues replaced (SLQPRAFQALIKEESPNC). This splice variant due to the elimination of 26 amino acids has 6 phosphorylation site removed that serve as regulatory domains important for MDM2-mediated ubiquitination (45). As a result, western blots revealed a more intense band for p53. Several p53 target genes of p53 were investigated from the RNA-seq data to help characterize the transcriptional action of the p53 alternative splice variant (Table 2). FoxM1 is typically repressed by wild-type (WT) p53 and amplified when p53 has a DNA-binding mutation (46, 47). Cdkn1a (or p21) is also positively regulated by p53 and mutation or loss of p53 results in a lack of induction (33, 48). Thbs1 is enhanced in the presence of WT p53 and p53 null cells compared to those harboring a missense mutation (49). In MOE^HIGH^, FoxM1 RNA was enhanced by 1.59-fold in the RNA-seq data, p21 was repressed by 1.98-fold, and Thbs1 was repressed 17-fold. Therefore, RNA expression for FoxM1, p21, and Thbs1 indicated that these genes were regulated by the p53AS in a similar manner to missense or gain-of-function mutations in p53. WT p53 induces MDM2 expression, while mutation typically results in poor or repressed activation of the MDM2 promoter, but no significant change was found at the RNA level of MDM2 (50). IGFBP3 has a possible 11 p53-responsive elements in the promoter and also in an intronic sequence that is frequently methylated, which can block p53 induction of this target (51). The MOE^HIGH^ that express a splice variant of p53 also expressed a 4.86 induction of the RNA (IGFBP3) transcript compared to MOE^LOW^. These results suggest that the p53AS has mixed functions acting more like WT p53 on some transcriptional targets, such as IGFBP3, and more like missense p53 on FoxM1, p21, and Thbs1 gene regulation.

**Table 2:** **p53 splice variant alters the mRNA expression of p53 target genes**.

Transcripts	MOE^LOW^	MOE^HIGH^	Fold change	FDR-adjusted *p*-value
Trp53 (p53) splice variant	79.62	111.81	1.40	0.0006
Foxm1	22.43	35.61	1.59	0.0002
Cdkn1a (p21)	158.98	80.15	−1.98	0.0002
Thbs1	281.28	16.52	−17.03	0.0002
Mdm2	64.97	61.03	1.28	0.5745
Igfbp3	16.65	80.93	4.86	0.0002

*RNA sequencing revealed expression of an alternatively spliced p53 transcript variant MOE^HIGH^ cells. Foxm1 was upregulated, Cdkn1a was repressed, and Thbs1 was repressed similar to regulation of these promoters by p53 containing a DNA-binding mutation. MDM2 regulation was unchanged, while Igfbp3 was upregulated similar to wild-type p53 action*.

### Key pathways altered in human HGSC are also modified in MOE^HIGH^

**Figure 5:**
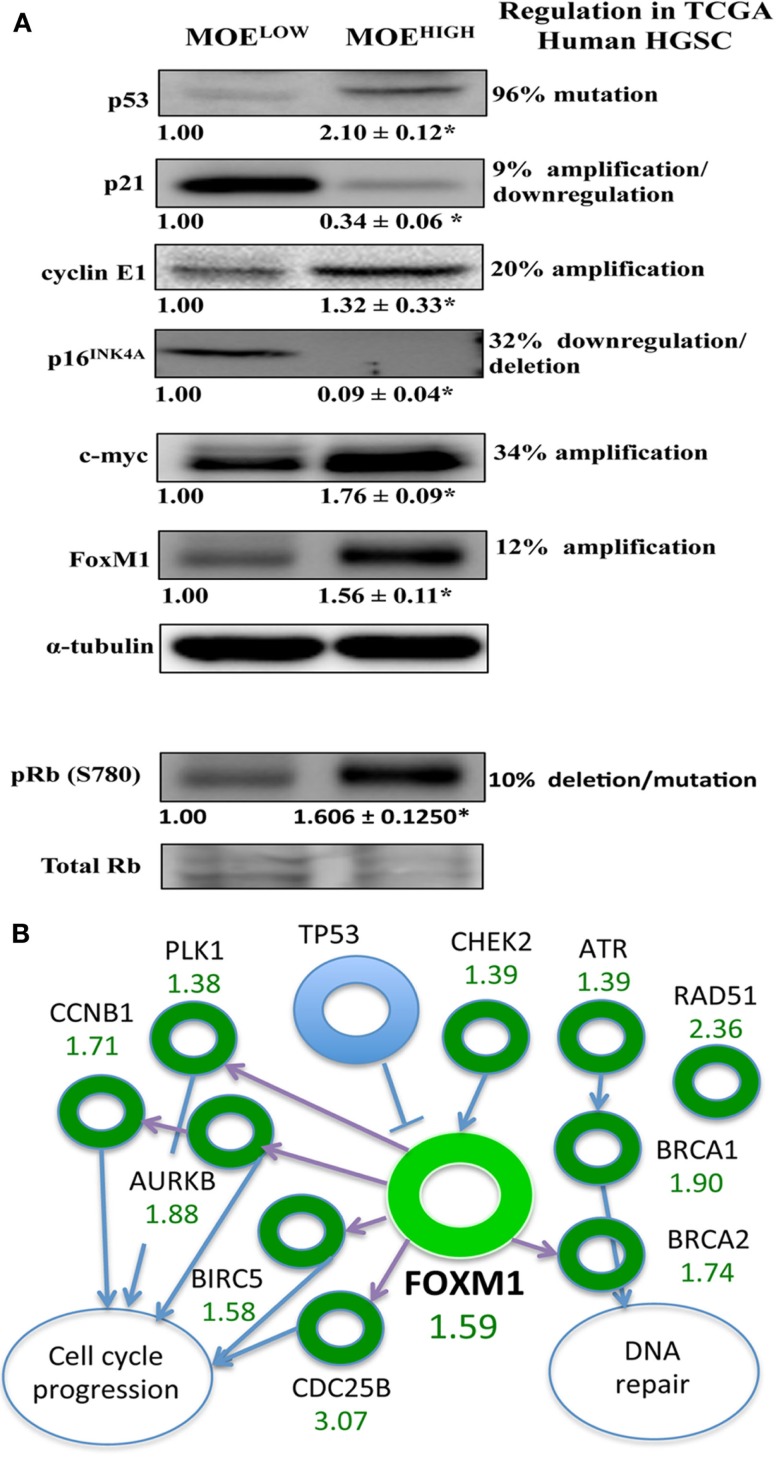
**MOE^HIGH^ cells have several pathways regulated similarly to human high-grade serous tumors**. **(A)** Western blotting analysis on MOE^HIGH^ and MOE^LOW^ cells probed for targets that have differential regulation in human tissue samples based on the TCGA database. p53 protein levels were elevated in MOE^HIGH^ cells due to overexpression of p53 splice variant. Cyclin E, pRB (S780), c-Myc, and FoxM1 expression levels were increased and p21 and p16^INK4A^ expression levels were decreased in MOE^HIGH^ compared to MOE^LOW^. Numbers under blots for Cyclin E, c-Myc, and FoxM1 represent fold change in densitometry between MOE^HIGH^ and MOE^LOW^ normalized to tubulin. Number under pRB (S780) blot represent fold change in densitometry between MOE^HIGH^ and MOE^LOW^ normalized to total RB. **(B)** FoxM1 pathway modified in MOE^HIGH^ similar to human high-grade serous tumors. Fold increase in RNA expression from MOE^HIGH^ compared to MOE^LOW^ from RNA-seq is shown in green.

The TCGA highlighted several players that were altered in a large percentage of human tissue samples. These targets were investigated using western blot in MOE^HIGH^ compared to MOE^LOW^ (Figure 5A). While the p53 DNA-binding domain was not mutated, the protein expression was higher in MOE^HIGH^. WT p53 induces p21 expression, yet p21 was repressed at the protein level, similar to human tumors that have a reduction due to mutation and/or downregulation in 7% of tumors. Cyclin E1 is amplified in about 20% of human HGSCs and the protein was elevated in the MOE^HIGH^ cells. Differential regulation of the Rb pathway was reported in TCGA data, and the MOE^HIGH^ showed an increase in phospho-Rb protein, a loss of p16INK4A protein, and amplified CCND1 transcript (RNA sequencing) (17). Lastly, c-myc and FoxM1 proteins were elevated in MOE^HIGH^ as compared to MOE^LOW^.

The FOXM1 pathway was amplified in roughly 90% of high-grade serous tumors according to the TCGA analysis (17). Since FoxM1 was amplified at an RNA and protein level, the pathways noted by the TCGA that were altered in human tissue samples were monitored in the RNA-seq results. The TCGA reported on the following transcripts that are regulated in the FOXM1 pathway, all of which (or their mouse homolog) were amplified in the TCGA data set and in the MOE^HIGH^ cell model: CHEK2, ATR, BRCA1, BRCA2, RAD51, CDC25B, BIRC5, AURKB, PLK1, and CCNB1 (Figure 5B).

### MOE^HIGH^ are resistant to DNA bulky strand adducting pathways

**Figure 6:**
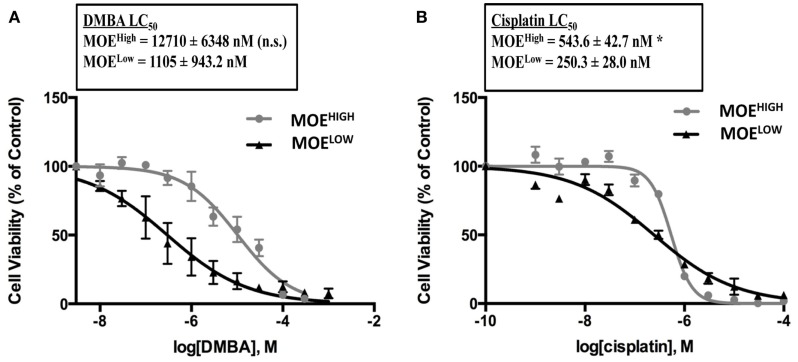
**MOE^HIGH^ cells display chemoresistance**. MOE^HIGH^ and MOE^LOW^ cells were treated with DMBA **(A)** and cisplatin **(B)**. Mean ± SEM *p-value ≤0.05 and not significant (n.s.).

One of the hallmarks of HGSC is the development of resistance to chemotherapy, and typically resistance occurs to carboplatin, although patients can also become resistant to paclitaxel (52). In order to determine if MOE^HIGH^ exhibited any differential response to chemotherapy, concentration-dependent growth curves were generated in response to the commonly used front-line therapies for ovarian cancer, cisplatin, and paclitaxel. The MOE^HIGH^ cells were less sensitive to cisplatin demonstrating an LC_50_ of 541 nM, which was almost double that of MOE^LOW^ (245 nM) (Figure 6B). Similarly, DMBA, which forms DNA adducts repaired by nucleotide excision repair, was able to induce cell death in MOE^LOW^ at an LC_50_ of 297 nM, while MOE^HIGH^ required 10.3 μM to induce death (Figure 6A). Interestingly, the cells displayed no significant differences in response to doxorubicin and paclitaxel (Figure S3 in Supplementary Material). Therefore, the MOE^HIGH^ transformed cellular model may also provide some insights into changes that allow oviductal-derived tumor cells to develop resistance to cisplatin and other bulky adducting compounds, like DMBA.

## Discussion

While several transgenic mouse models and engineered cell models have demonstrated that oviductal epithelium can form HGSC, a spontaneous model of tumor formation from the oviduct has not yet been reported. Spontaneous models of OSE after continuous passage have been used for many studies including studies that help to understand the progression of cancer and the properties of the cells, such as aneuploidy, transcriptional alterations, enhanced stem-cell like properties, response to chemotherapy, and as a syngeneic model (26, 28). Continuous passaging of primary, non-tumor forming CD1 oviductal cells revealed that they became more secretory dominated in culture, proliferated more rapidly, formed 2D foci and soft agar colonies. RNA profiling revealed several pathways modified in the cellular model that were also altered in HGSC, such as FoxM1. Similar to models derived from OSE, the tumors were mostly undifferentiated, but they expressed oviductal markers. The MOE^HIGH^ cells demonstrated a resistance to cisplatin and DMBA, but not to doxorubicin, H_2_O_2_, and paclitaxel. Overall, these studies provide a unique cellular model that can be used to monitor the changes that accumulate over time to produce a spontaneous tumor from oviductal cells exposed to continuous passaging.

MOE^HIGH^ represent the first spontaneous cellular model of cancer derived from an oviductal cell line. This model serves as a complimentary model system to several similar ones derived from the OSE of rats and mice (25, 28, 53–55). While all of these models are tumorigenic, almost all of them produced undifferentiated tumors, similar to the MOE^HIGH^ in this study. Only recently, a spontaneous OSE model was generated that exhibited not only phenotypic aspects of HGSC but also molecular ones (28). MOE^HIGH^ while lacking expression for several common immunohistochemical markers for HGSC, such as cytokeratin 8, did express Pax8 and oviductal glycoprotein confirming their oviductal origin. MOE^HIGH^ is also the first spontaneous model from an outbred strain, as previous OSE models have been generated from C57Bl/6 or FVB strains. MOE^HIGH^ could potentially be grafted into immune competent hosts, but difficulty has been reported in the literature for allografting into outbred hosts, such as CD1 mice (56). The enhanced genetic diversity on an outbred strain may provide a better tool to uncovering mechanisms behind spontaneous tumor formation.

The most common mutation in human HGSC is in p53, typically found in the DNA-binding domain. While MOE^HIGH^ cells did not display a standard DNA-binding mutation, the protein was alternatively spliced such that it lacks negative c-terminal regulatory domains. Interestingly, this splice variant could function similarly to truncation mutations found in the c-terminus of the human gene, which was demonstrated to lack growth arrest and apoptotic functions (57). Ceramide, an inhibitor of SRSF1 splicing activity, was recently shown to restore WT p53 function and could be a potential way to re-activate WT p53 in “deletion” mutated cells (58). Given that the model is of murine origin, and rodents have been shown to have unique splicing of p53, certain aspects of this model may be species specific (59). In addition, the MOE^HIGH^ cellular model did not demonstrate any mutations in BRCA1 or 2, but consistent with human disease, had amplification of cyclin E1. Human tumors exhibit a mutual exclusivity between BRCA mutations and cyclin E amplifications (60). MOE^HIGH^ did recapitulate some of the pathway modifications typically seen in HGSC, such as amplification of FoxM1 and its targets, amplification of the c-myc protein, cyclin E, reduced Rb function, and a loss of p16INK4a. This is important because most of the original evidence supporting the origin of HGSC came from women with mutations in BRCA1/2 or from genetically engineered mouse models with BRCA1/2 floxed alleles deleted in fallopian (oviductal) cells. The MOE^HIGH^ model demonstrates that in the absence of BRCA mutation and/or loss, those oviductal cells can become tumorigenic, and recapitulate many of the dysfunctional pathways associated with HGSC.

RNA sequencing of MOE^HIGH^ cells revealed the important biological processes or pathways that were modified included splicing, WNT signaling, and DNA synthesis. While DNA synthesis is not surprising given the high rate of proliferation, pathway modification of splicing, and WNT are consistent with findings from HGSC and represent possible therapeutic targets (61–63). WNT signaling is essential in the developmental origins of the oviductal cells, and the dual role of many developmental factors is linked with tumor formation. HGSC has a variety of WNT factors dysregulated including Wnt5a, Wnt11, Wnt7a, and Sox7 (64–66), and inhibition of certain WNT targets, such as GSK3β, has shown efficacy in preclinical xenograft models (67). While several WNT pathway members were downregulated, such as Fzd2, 4, 5, and 8, soluble ligands, such as WNT4, and target genes, such as cyclin D1 and Myc, were amplified. Interestingly, a recently defined spontaneous ovarian tumor model from the OSE also displayed changes in Wnt signaling, suggesting that regardless of cellular origin, certain pathways may provide ideal new drug targets for HGSC cancers (28).

The MOE^HIGH^ cellular model did not form peritoneal disease, and was only able to form tumors in the subcutaneous space, which was reflected in a downregulation of “pathways in cancer” in the KEGG results where most of the genes were metastatic or tumor suppressors. Perhaps, the cellular model does not form widely disseminated tumors because key pathways in ovarian cancer were not disrupted, such as BRCA or PTEN. Another possible explanation is that there are key signaling molecules that block peritoneal dissemination that are still present in the cells, and perhaps uncovering these will provide new targets to exploit for reducing peritoneal spread. One such pathway that was still present in the cells was Pax2 (data not shown). Pax2 is typically lost in early lesions of HGSC, and studies have demonstrated that re-expression of Pax2 in HGSC can induce cellular death (68). Several other *in vitro*-derived models of fallopian or oviductal cancer have primarily been xenografted into SCID mice, and perhaps the difference in background strain influences the spread of the disease. Lastly, xenografting directly into the ovarian bursa may increase the chance of developing an early stage tumor that may be required before subsequent peritoneal spread.

In summary, a spontaneous model of oviductal cancer was derived by continuously passaging using an outbred CD1 murine cell line that developed features of HGSC, such as amplification of FOXM1 and c-myc, resistance to cisplatin, and loss of Cdkn2a. The model produced tumors only in the s.c. compartment and may represent early disease or a model that lacks key regulators of peritoneal spread. Further investigation is warranted to understand the role of splicing and WNT signaling in this model system. This model offers the opportunity to uncover a step-wise progression of tumor formation from an oviductal origin to be compared to human disease, derived from possibly both the ovarian surface and the oviduct.

## Conflict of Interest Statement

The authors declare that the research was conducted in the absence of any commercial or financial relationships that could be construed as a potential conflict of interest.

## Supplementary Material

The Supplementary Material for this article can be found online at http://journal.frontiersin.org/article/10.3389/fonc.2015.00154

## Footnotes

^1^http://tophat.cbcb.umd.edu

^2^http://cufflinks.cbcb.umd.edu/
