# Supplementary material for: Spontaneous Transformation of Murine Oviductal Epithelial Cells: A Model System to Investigate the Onset of Fallopian-Derived Tumors
Source: Front Oncol. 2015 Jul 17;5:154. doi: 10.3389/fonc.2015.00154 (PMC4505108; doi:10.3389/fonc.2015.00154)
Supplement: Supplementary file 1 [file image_1.pdf]

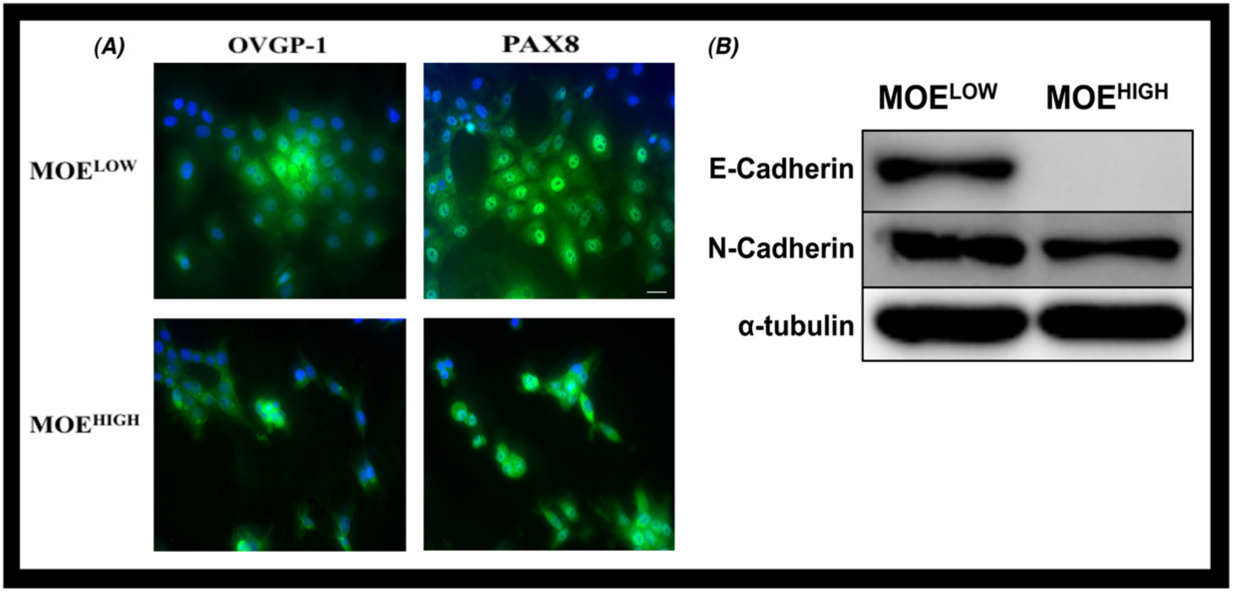

Supplementary Figure S1 | Immunofluorescence demonstrating that MOE^{HIGH} and MOE^{LOW} express Pax8 and OVGP1 and using western blot the MOE^{HIGH} no longer express E-cadherin.
