# Supplementary material for: Spontaneous Transformation of Murine Oviductal Epithelial Cells: A Model System to Investigate the Onset of Fallopian-Derived Tumors
Source: Front Oncol. 2015 Jul 17;5:154. doi: 10.3389/fonc.2015.00154 (PMC4505108; doi:10.3389/fonc.2015.00154)
Supplement: Supplementary file 2 [file image_2.pdf]

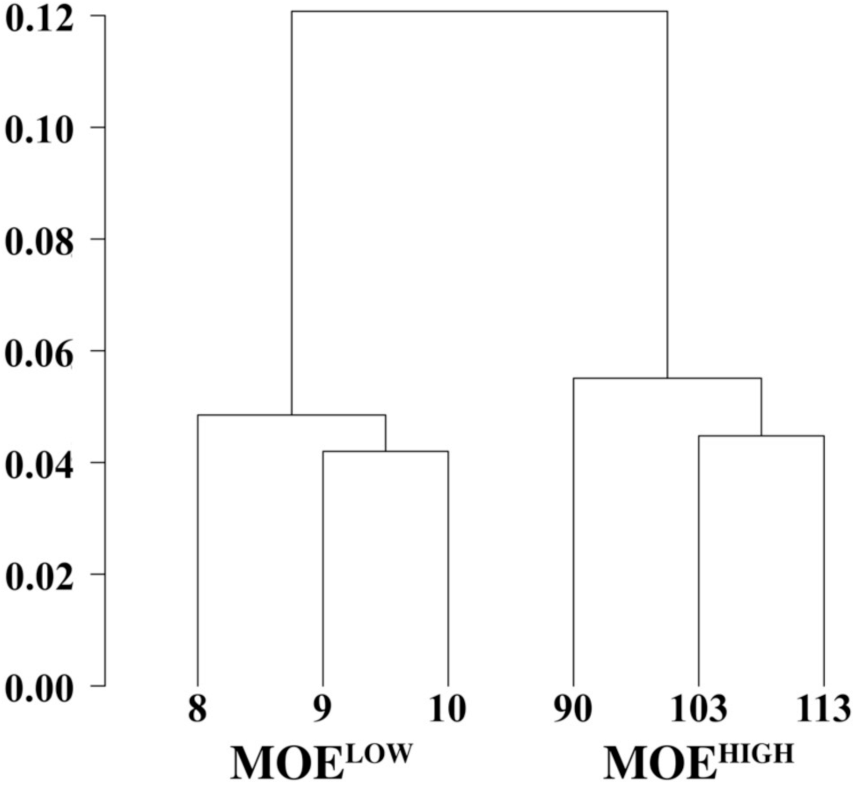

Supplementary Figure S2 | Dendrogram demonstrating that MOE^{HIGH} and MOE^{LOW} samples clearly separate and segregate together based on RNA sequencing analysis.
